# Monocyte Chemotactic Protein-1, Fractalkine, and Receptor for Advanced Glycation End Products in Different Pathological Types of Lupus Nephritis and Their Value in Different Treatment Prognoses

**DOI:** 10.1371/journal.pone.0159964

**Published:** 2016-07-26

**Authors:** Lan Lan, Fei Han, Xiabing Lang, Jianghua Chen

**Affiliations:** Kidney Disease Center, The First Affiliated Hospital, College of Medicine, Zhejiang University, Hangzhou, 310003, P.R. China; University of Utah School of Medicine, UNITED STATES

## Abstract

**Background:**

Early diagnosis is important for the outcome of lupus nephritis (LN). However, the pathological type of lupus nephritis closely related to the clinical manifestations; therefore, the treatment of lupus nephritis depends on the different pathological types.

**Objective:**

To assess the level of monocyte chemotactic protein (MCP-1), fractalkine (Fkn), and receptor for advanced glycation end product (RAGE) in different pathological types of lupus nephritis and to explore the value of these biomarkers for predicting the prognosis of lupus nephritis.

**Methods:**

Patients included in this study were assessed using renal biopsy. Class III and class IV were defined as the proliferative group, class V as non-proliferative group, and class V+III and class V+IV as the mixed group. During the follow-up, 40 of 178 enrolled patients had a poor response to the standard immunosuppressant therapy. The level of markers in the different response groups was tested.

**Results:**

The levels of urine and serum MCP-1, urine and serum fractalkine, and serum RAGE were higher in the proliferative group, and lower in the non-proliferative group, and this difference was significant. The levels of urine and serum MCP-1 and serum RAGE were lower in the poor response group, and these differences were also significant. The relationship between urine MCP-1 and urine and serum fractalkine with the systemic lupus erythematosus disease activity index was evaluated.

**Conclusion:**

The concentration of cytokines MCP-1, fractalkine, and RAGE may be correlated with different pathology type of lupus nephtitis. Urine and serum MCP-1 and serum RAGE may help in predicting the prognosis prior to standard immunosuppressant therapy.

## Introduction

One of the causes of SLE may be an imbalance in the activation and suppression of immune function. Lupus nephritis (LN) is a multifactorial disease and involves both genetic and environmental causes as suggested by some studies. For example, glomerular microthrombosis may be partly caused by an epistatic effect of the PAI-1 and FGB genes [1]. We also found that, a variety of cytokines and cytokine receptors are involved in the regulation of immune function [2,3].

Early diagnosis is important for the outcome of lupus nephritis. However, the pathological type of lupus nephritis is closely related to its clinical manifestations, the treatment of lupus nephritis depends on the different pathological types. The “gold standard” for pathological diagnosis depends on an invasive kidney biopsy. Different types of lupus nephritis have different characteristics of immunity and inflammatory activation. Currently, morphology is the main indicator of pathology. The biomarkers such as urine and serum inflammatory factors, which reflect the different inflammatory characteristics of lupus nephritis, can be tested. Testing of these markers not only helps to distinguish the pathological type, but may also be beneficial for recognizing the different inflammatory symptoms of lupus nephritis

Monocyte chemoattractant protein (monocyte chemotactic protein 1, MCP-1) primarily targets monocytes and T lymphocytes and induces the production of inflammatory mediators, such as interleukin (IL)-1 and IL-6, and it also injures tissues through enhanced monocyte-macrophage cell adhesion [4,5] Epidemiological studies that address mutations in the CCL2 (MCP-1) gene support the hypothesis that CCL2 mediates renal inflammation [6].

Fractalkine (Fkn, CX3CL1) is a member of the CX3C-chemokine family that is expressed as both soluble and transmembrane/mucin hybrid forms, thus combining chemoattractant functions together with receptor/adhesion molecule properties [7]. Fractalkine may be expressed in crescentic glomerulonephritis and acute allograft rejection in kidney transplantation [7,8]. Fractalkine may mediate critical physiological functions during immune regulation[7,9]. in its soluble forms, fractalkine mediates the chemotaxis of immune cells, and the membrane-bound form of fractalkine may mediate leukocyte capture and infiltration via its role as an adhesion molecule [9].

RAGE (receptor for advanced glycation end products), a multi-ligand receptor that belongs to the immunoglobulin superfamily of transmembrane proteins, may expand and intensify the immune response [10]. RAGE expression is prominent on the activated endothelium, where it mediates leukocyte adhesion and the transmigration of SMCs [11]. RAGE plays an important role in the inflammation.

Limited data are available about the urine and serum levels of MCP-1, fractalkine, and RAGE for different pathological types of lupus nephritis. The urine and serum levels of these three potential markers were determined, and the inflammatory symptoms of different types of lupus nephritis were determined. The concentration of different markers was assessed in patients with lupus nephritis with different treatment response.

## Materials and Methods

### Study subjects

The study protocols conformed to the provisions of the Declaration of Helsinki. The Ethics Committee of the First Affiliated Hospital, College of Medicine, Zhejiang University, China, approved the protocol and informed consent was obtained from all patients. The participants had provided their written informed consent before the renal biopsy, and they had agreed to stay the urine and serum specimen for scientific research before and after renal puncture and during the outpatient follow-up.

All patients with SLE who underwent renal biopsy and were followed-up at the Kidney Disease Center between June 2004 and June 2013 were retrospectively analyzed. The diagnosis of SLE was in accordance with the 1997 American College of Rheumatology criteria [12]. Renal pathology was classified according to the 2003 standardized International Society of Nephrology/Renal Pathological Society classification [13]. Patients who met the following criteria were included in the analysis: (1) patients who were diagnosed with lupus nephritis at the center between June 2004 and June 2013; (2) all patients who underwent renal biopsy and whose serum and urine specimens before biopsy were available; and (3) patients who did not use immunosuppressive agents within the preceding 3 months except for glucocorticoids. The following patients were excluded from the study: (1) patients who were younger than 18 years or >60 years; and (2) patients with severe infection or with other immune system diseases.

SLE disease activity was calculated using the Safety of Estrogens in Lupus Erythematosus National Assessment-Systemic Lupus Erythematosus Disease Activity Index (SLEDAI). The activity index (AI) and chronicity index (CI) were also calculated [14]. Histological parameters were used to calculate composite scores for the AI and CI, which were cellular crescents, fibrinoid necrosis, karyorrhexis, endocapillary proliferation, leukocytic exudation, hyaline deposits, and interstitial infiltration. Each feature present contributed a score of 3, except fibrinoidnecrosis and crescents, which were weighted twice as much; hence, the maximum possible score was 24.

Using renal pathology and the 2003 standardized International Society of Nephrology/Renal Pathological Society classification, patients were classified from class I-VI in the present study. Class III and class IV were defined as the proliferative group, class V was defined as the non-proliferative group, and class V+III and class V+IV were defined as the mixed group.

### Biomarker assays

All patients underwent routine laboratory assessments. Urine and serum samples were collected at baseline from all patients with lupus nephritis. Blood samples were obtained for determining the complete blood cell count, serum creatinine, serum C3, C4, and anti-ds-DNA. Urine spot samples were obtained from patients’ morning urine, which was stored at -70°C until the experiments were performed. Before testing, the samples was brought to room temperature. All patients, regardless of severity of the disease, were asked to provide 24-h urine for determining proteinuria. Patients were advised to pass urine at 08:00 am and to collect all urine subsequently till 08:00 am the next morning (24-h period). The adequacy of urine collection was determined using creatinine excretion. Each sample was tested in duplicate.

Urine was tested using enzyme-linked immunosorbent assay (ELISA) kits for RAGE, MCP-1, and fractalkine; the kits were purchased from R&D Laboratories (Minneapolis, MN) and were used as indicated by the manufacturer. Human MCP-1 Duo Set, catalog number: DCP300; Human fractalkine Duo Set: DCX3l0; and urine creatinine assay kit, number: ab65430 were used.

### Biostatistical analysis

All the experimental data were analyzed using the (SPSS, Inc, Chicago, IL, USA) software. Experimental data from the ELISA were expressed as the mean±standard error, unless otherwise specified. Urine specimens were corrected using urine creatinine. Groups were compared against each other using the Mann-Whitney U test, and Kruskal-Wallis test was used in more than two samples. The least significant difference method was used for multiple comparisons between different groups. Logistic regression was used to assess the value of the markers in predicting the prognosis. Correlation analysis analysis was used to assess whether SLEDAI, AI, or CI were related markers.

## Results

From June 2004 to June 2013, 197 patients underwent renal biopsy and were diagnosed with lupus nephritis including 71 patients with class IV lupus nephritis, 53 patients with class V lupus nephritis, 33 patients with class V+IV lupus nephritis, 11 patients with class III lupus nephritis, 5 patients with class II lupus nephritis, 3 patients with class I lupus nephritis, 10 patients with class V+III lupus nephritis, 11 patients who could not be distinguished by slight pathological changes. According to the criteria of the present study, 82 patients with class III and class IV lupus nephritis were included as the as proliferative group, 53 patients with class V lupus nephritis were the non-proliferative group, and 43 patients with class V+III and class V+IV lupus nephritis were the mixed group, as shown in Table 1.

**Table 1:** Baseline status of proliferative, non-proliferative, and mixed groups.

Group	proliferative LUPUS NEPHRITIS N = 82	non-proliferative LUPUS NEPHRITIS N = 53	Mixed LUPUS NEPHRITIS N = 43	P value
Sex (female/male)	72/10	43/10	35/8	0.49
Year (year)	35.36 ± 11.76	39.73 ± 12.75	37.93 ± 12.70	0.19
Lupus nephritis time (month)	11.40 ± 22.0	10.90 ± 23.20	12.10 ± 24.10	0.48
24h urine protein (g)	4.19 ± 2.76	4.34 ± 2.71	5.15 ± 2.62	0.13
creatinine (μmol/L)	104.48 ± 64.94	90.86 ± 76.58	103.28 ± 94.73	0.24
Serum albumin (g/dl)	27.09 ± 7.67	26.42 ± 9.06	24.43 ± 6.45	0.15
White blood cells (10E9/L)	5.43 ± 2.87	6.38 ± 3.45	5.49 ± 2.99	0.12
Hemoglobin (g/L)	97.56 ± 17.71	105.61 ± 19.05	101.02 ± 23.81	0.11
Platelet (10E9/L)	159.23 ± 80.48	176.57 ± 64.99	164.74 ± 75.42	0.24
Complement C3 (ng/L)	52.72 ± 23.89	54.54 ± 19.67	50.29 ± 25.66	0.08
Complement C4 (ng/L)	10.01 ± 7.31	12.58 ± 7.42	11.92 ± 10.16	0.07
AI score	8.06 ± 1.72	7.81 ± 2.15	8.12 ± 1.41	0.11
CI score	4.33 ± 0.81	4.62 ± 1.05	4.56 ± 1.37	0.10
SLEDAI score	12.73 ± 5.02	10.81 ± 4.95	11.21 ± 4.85	0.08

During the follow-up, 40 of the 178 enrolled patients were confirmed as having a poor response to standard immunosuppressant therapy (patients who could not achieve complete/partial remission at the end of 3 months with the standard immunosuppressant therapy of prednisone +cyclophosphamide.), as shown in Table 2.

**Table 2:** The baseline status of different response to the immune therapy.

Group	Poor response	Good response	P value
Sex (female/male)	30/10	115/23	0.22
Year (year)	35.54 ± 8.91	37.86 ±1 2.67	0.15
Lupus nephritis time (month)	10.35 ± 21.84	11.65 ± 22.10	0.20
24 h urine protein (g)	6.57 ± 3.28	4.95 ± 2.72	0.37
Serum albumin (g/dl)	24.41 ± 8.09	27.82 ± 7.74	0.17
creatinine (μmol/L)	112.89 ± 59.34	101.01 ± 101.42	0.47
White blood cells (10E9/L)	6.63 ± 3.79	5.48 ± 2.83	0.15
Hemoglobin (g/L)	104.60 ± 23.93	106.91 ± 67.29	0.65
Platelet (10E9/L)	162.91 ± 79.22	163.34 ± 71.72	0.94
Complement C3 (ng/L)	46.92 ± 24.90	50.02 ± 22.85	0.53
Complement C4 (m(ng/ml)	10.03 ± 5.77	11.15 ± 7.03	0.30
AI score	7.89 ± 1.97	7.44 ± 1.31	0.52
CI score	5.02 ± 1.35	4.48 ± 1.02	0.39
SLEDAI score	12.15 ± 4.96	11.18 ± 4.98	0.29
Pathology type (%)			
III	0	11 (7.0)	0.13
IV	18 (45)	53 (33.7)	0.62
V	11 (27.5)	42 (26.7)	0.94
IV+V	8 (20.0)	25 (15.9)	0.62
V+III	3 (7.5)	7 (4.4)	0.54

### Levels of MCP-1, fractalkine, and RAGE in different pathological types

We summarized the levels of biomarkers for different pathological types in Table 3.

**Table 3:** Levels of biomarkers in the proliferative, non-proliferative, and mixed groups.

	Urine MCP-1(pg/ml creatinine)	Serum MCP-1 (pg/ml creatinine)	Serum Fkn (ng/mmol creatinine)	Serum Fkn(ng/mmol creatinine)	Serum RAGE(pg/ml)
proliferative group	1240.65±876.38	354.49±598.60	4.44±3.05	2.53±1.24	2020.74±1421.27
nonproliferative group	544.47±430.63	200.40±171.83	2.37±1.88	2.07±1.19	1257.94±862.83
mixed group	595.20±603.42	273.31±429.31	3.42±2.45	2.28±1.05	1707.85±1383.43

In the proliferative group, the urine and serum MCP-1 levels were 1240.65±876.38 pg/ml creatinine and 354.49±598.60 pg/ml, respectively. Urine and serum fractalkine levels in the proliferative group were 4.44 ± 3.05 ng/mmol creatinine and 2.53 ± 1.24 ng/ mL, respectively. The serum and urine RAGE levels were examined; however, the urine RAGE concentration was below the limit of detection. The serum RAGE level was tested and was 2020.74 ± 1421.27 pg/ml in the proliferative group.

In the non-proliferative group, the urine and serum MCP-1 were 544.47 ± 430.63 pg/ml creatinine and 200.40 ± 171.83 pg/mL, respectively. The urine and serum fractalkine levels were 2.37 ± 1.88 ng/mmol creatinine and 2.07 ± 1.19 ng/mL, respectively. The serum RAGE level was 1257.94 ± 862.83 pg/mL.

**Fig 1:**
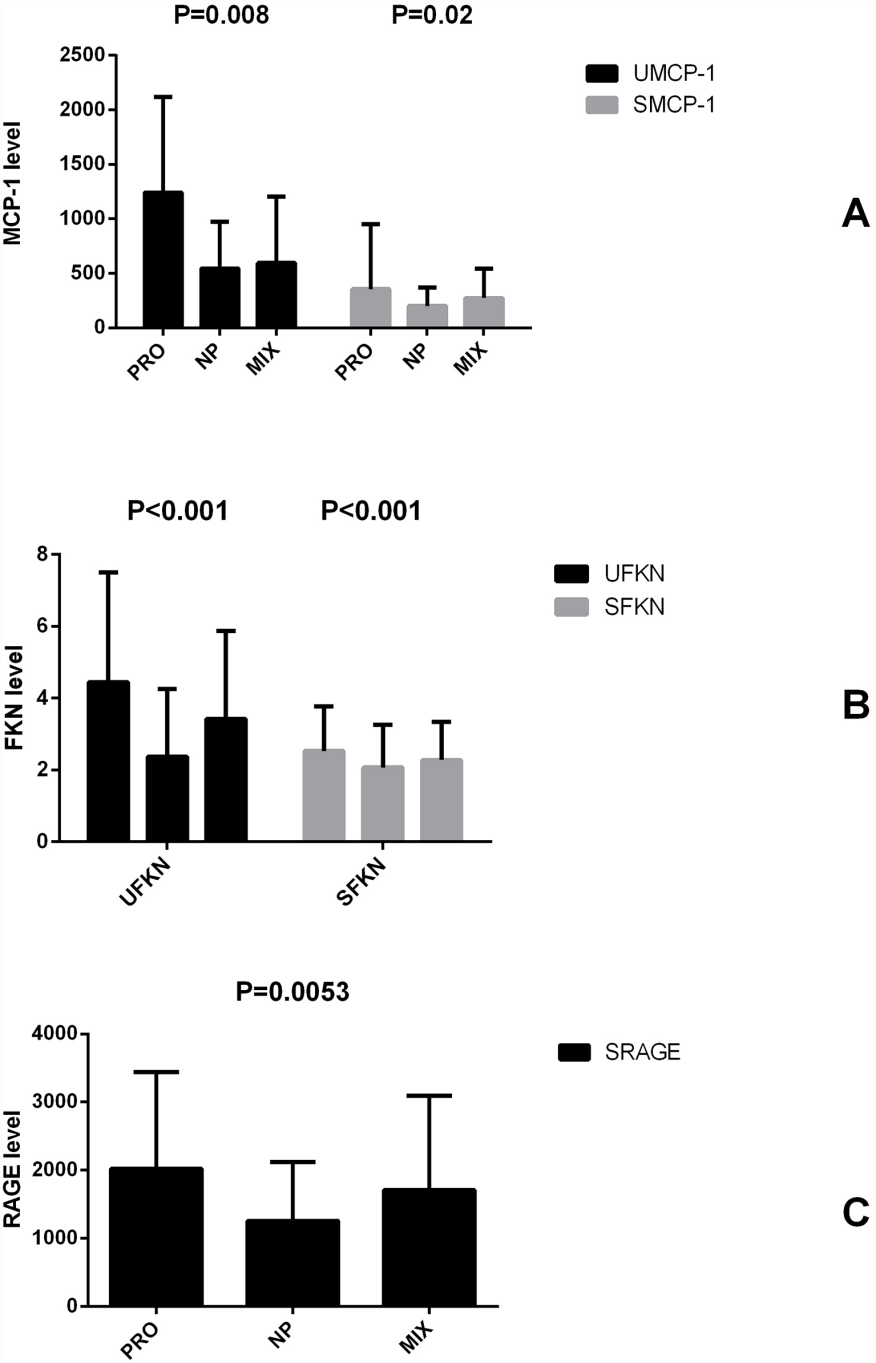
MCP-1, fractalkine and RAGE had different levels for the different pathological types of lupus nephritis.

In the mixed group, the urine and serum MCP-1 levels were 595.20 ± 603.42pg/ml creatinine and 273.31 ± 429.31 pg/mL, respectively. The urine and serum fractalkine levels were 3.42 ± 2.45 ng/mmol creatinine and 2.28 ± 1.05 ng/mL, respectively. The serum RAGE level was 1707.85 ± 1383.43 pg/mL. The MCP-1, fractalkine and RAGE levels indicated different levels for different pathological types of lupus nephritis, and the difference in the levels of urine and serum MCP-1 in the two groups was significant (urine MCP-1: *P* = 0.008; serum MCP-1: *P* = 0.02; urine Fkn: *P* = 0.02; serum Fkn: *P* = 0.006; serum RAGE: *P* = 0.0053). Significant differences among the three groups are summarized in Fig 1.

### Level of MCP-1, fractalkine, and RAGE in groups with different responses to immunosuppressant therapies

We summarized the level of biomarkers for the different responses to immunosuppressant therapies in Table 4.

**Table 4:** Level of biomarkers in the good response to immunosuppressant therapy group and the poor response to immunosuppressant therapy group.

	UrineMCP-1(pg/ml creatinine)	Serum MCP-1 (pg/ml creatinine)	Urine Fkn (ng/mmol creatinine)	Serum Fkn((ng/mmol creatinine)	Serum RAGE(pg/mL.)
good response to immunosuppressant therapy group	943.47 ± 791.69	352.75 ± 535.71	3.43 ± 2.80	2.61 ± 2.40	1700.42 ± 1345.38
poor response to immunosuppressant therapy group	483.95.33 ± 433.21	180.81 ± 105.94	3.89 ± 2.40	2.23 ± 2.80	1141.64 ± 828.53

**Fig 2:**
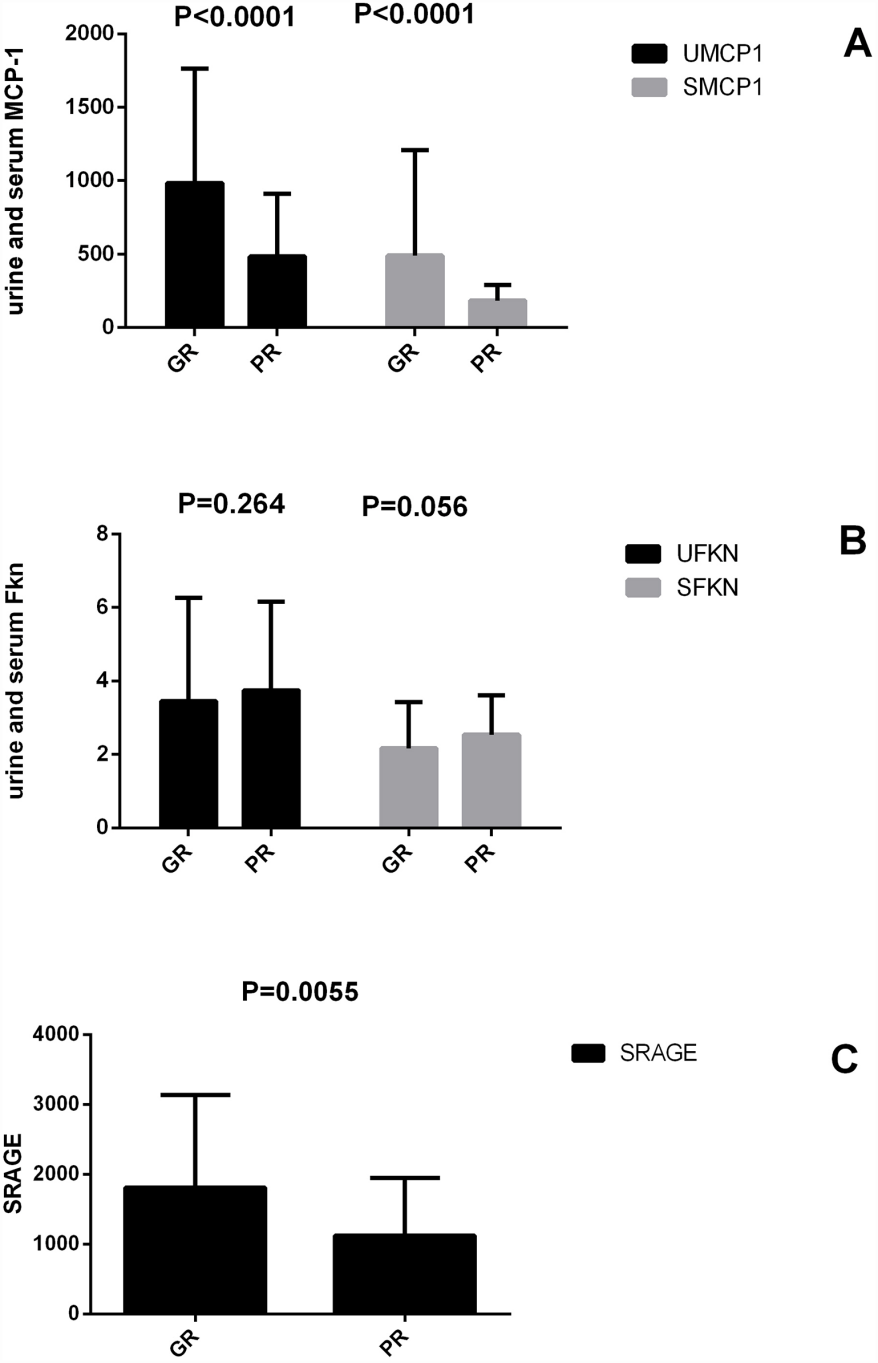
Biomarkers showed different responses to standard immunosuppressant therapy.

In the poor response to immunosuppressant therapy group, the urine MCP-1 level was 483.95.33 ± 433.21 pg/ml creatinine, and the serum MCP-1 level was 180.81 ± 105.94 pg/ml. In the good response to immunosuppressant therapy group, the urine and serum MCP-1 levels were 943.47 ± 791.69 pg/ml creatinine and 352.75 ± 535.71 pg/mL, respectively. The serum RAGE level in the poor response to immunosuppressant therapy group was 1141.64 ±8 28.53 pg/ml, and in the good response to immunosuppressant therapy group, it was 1700.42 ± 1345.38 pg/mL. The difference in the levels of urine and serum MCP-1 in the two groups was significant (*P*<0.0001; *P*<0.0001). The difference in the levels between serum RAGE in the different groups was also significant (*P* = 0.0055). The urine and serum fractalkine levels in groups with different responses to immunosuppressant therapy were not significantly different. The significant differences between the two groups are summarized in Fig 2. Logistic regression was used to assess the value of markers for predicting the prognosis of lupus nephritis. The significance level of urine and serum MCP-1 levels was *P* = 0.002 and *P* = 0.048, respectively, the significance level of urine and serum Fkn levels was *P* = 0.264 and *P* = 0.056, respectively, and the significance level of serum RAGE was *P* = 0.043. According to the logistic regression, lower urine and serum MCP-1 and serum RAGE may be independent risk factors of a poor response to immunosuppressant therapy.

### Correlation analysis of MCP-1, fractalkine, and RAGE with SLEDAI, AI, and CI

**Fig 3:**
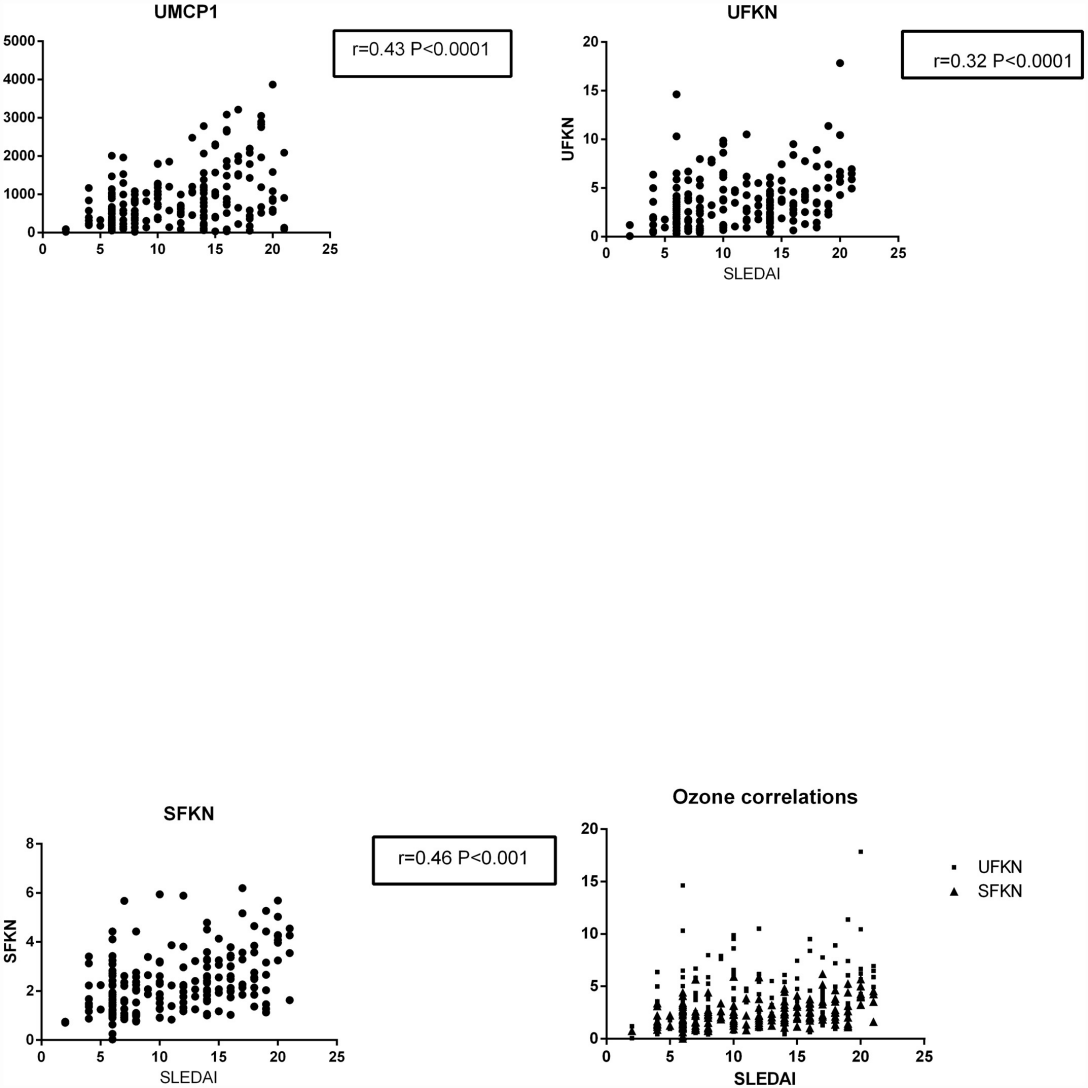
Urine MCP-1 and urine and serum fractalkine levels related to the SLEDAI.

The correlation analysis between the urine MCP-1 level and SLEDAI showed an r = 0.43 (95% confidence interval: 0.2970–0.5419, *P*<0.0001); however, the analysis between serum MCP-1 and SLEDAI did not show any significant correlation (r = 0.10, 95% confidence interval: -0.04606 to 0.2476, *P* = 0.1749). The related analysis between the urine fractalkine and SLEDAI showed an r = 0.32 (95% confidence interval: 0.1543–0.4187, *P*<0.0001), and the analysis between the serum fractalkine and SLEDAI showed an r = 0.46 (95% confidence interval: 0.2800–0.5268, *P*<0.0001). The analysis between the serum RAGE and SLEDAI showed an r = 0.12 (95% confidence interval: -0.02454 to 0.2653, *P* = 0.11). The relationship between urine and serum MCP-1, urine and serum Fkn, and serum RAGE with AI and CI were also analyzed, but the results were not significant. The correlation analysis is shown in Fig 3.

## Discussion

This study found that these markers were at different levels for different pathological types. All urine and serum MCP-1 levels showed a higher concentration in the proliferative group and a lower concentration in the non-proliferative group. The level of urine and serum MCP-1 in the mixed group was between the two other groups. Similar to MCP-1, the urine and serum fractalkine and serum RAGE also showed similar results for different pathological types of lupus nephritis.

These markers could predict the response to immunosuppressant therapy. In the present study, the lower urine and serum MCP-1 and serum RAGE levels could predict the poor response to immunosuppressants prior to therapy. However, the urine and serum fractalkine levels did not provide any advantage for predicting the prognosis of therapy. The urine MCP-1 and urine and serum fractalkine were related to the SLEDAI, but a relationship between AI and CI with all markers was not found.

In studies on autoimmune disease, MCP-1 was shown to be associated with the proliferative reaction. Studies have indicated that cell-surface proteins are unregulated during periods of inflammation. Pro-inflammatory cytokines, such as TNF-α, IL-1, and MCP-1 may upregulate leukocyte adhesion molecules via a nuclear factor-kappaB (NF-κB)-dependent process [15]. Campbell et al also showed that MCP-1 helps in the pathogenesis of inflammation and systemic autoimmune disease via TWEAK/TWEAK receptor signaling [16].

Recently, atherosclerosis has also been regarded as an inflammatory disease. During vascular atherosclerosis, the CX3CL1/CX3CR1 chemokine participates in the process of atherosclerotic pathology [17]. Stolla et al found that fractalkineis expressed at all stages of atherosclerotic lesion formation [18]. Studies also suggested that fractalkine stimulates cells growth in rheumatoid arthritis-fibroblast-like synoviocytes (RA-FLS) and that an NF-κB pathway blocker inhibits fractalkine, thus promoting the proliferation of RA-FLS. Fractalkine induced the activation of NF-κB activity. Fractalkine up regulates fractalkine mRNA expression in RA-FLS via the NF-κB pathway [19].

In diabetic mice, Chen et al [20] found that in vivo, RAGE^-/-^ mice correlated with the reduced proliferative responses of RAGE^-/-^ T cells in mixed leukocyte reactions and in wild-type T cells cultured with TTP488. In patients with RA, HMGB1 upregulated the expression of TLR4 and RAGE on the surface of synovial fluid in RA and was dependent on the activation of p38 mitogen-activated protein kinases and NF-κB [21].

This study found that all tested markers had higher expression in the proliferative lupus nephritis group and showed lower expression in the non-proliferative lupus nephritis group, which not only furthers the understanding of different characteristics of lupus nephritis but also may help in diagnosing the type of lupus nephritis before biopsy and during follow-up. All markers studied were related to inflammatory mechanisms, or partially participate in the NF-κB pathway. This result reveals that proliferative lupus nephritis is related to the NF-κB pathway. The study by Ling Zheng [22] found that in patients with type IV lupus nephritis, the tubules and meningeal cells showed higher NF-κB p65 and p60 protein expression than that of patients with non-proliferative lupus nephritis, which is also consistent with the present study.

The relationship between SLEDAI and markers was evaluated in this study. Many studies showed that cytokine markers could predict the relapse of SLE and that they are always related to the activity of lupus nephritis. The study by Aizawa et al [23] found that the measurement of urine fractalkine and MCP-1 concentrations may be useful as a noninvasive method for predicting the disease activity of glomerulonephritis in children. Martens et al [24] also found that RAGE is likely associated with the severity of disease and the initial response to treatment in lupus nephritis. The present study further improved these results, which may help in judging the severity of disease.

In the present study, patients with lupus nephritis with poor efficiency did not reach complete remission/partial remission with prednisone 0.8–1.0 mg/kg+cyclophosphamide 1 g/m^2^ ×3 m, and the urine and serum MCP-1 and serum RAGE showed lower levels in the patients with poor efficiency. As previous studies reported, the standard dose of prednisone blocks the expression of NF-κB [25,26]. Researchers also found that immunosuppressants CYC and bortezomib may act on the NF-κB pathway [27]. Based on the present study, the urine and serum MCP-1, serum fractalkine, and serum RAGE may predict the prognosis of patients with lupus nephritis before therapy. Patients with lower marker levels may be prone to a poor prognosis with standard treatment; therefore, multi-target therapy should be more effective. The reason why lower level of markers in patients correlates with a poor prognosis remains unclear, but it is correlated with expression of the NF-κB pathway in different patients with lupus nephritis.

Finally, a longer follow-up period and an increase in patient enrollment are necessary to further identify and validate markers for lupus.

## Supporting Information

S1 Data Data xlsx file. (XLSX)
